# Three new species of *Paraboea* (Gesneriaceae) from limestone karsts of China based on morphological and molecular evidence

**DOI:** 10.1186/s40529-017-0207-5

**Published:** 2017-12-01

**Authors:** Wei-Bin Xu, Jing Guo, Bo Pan, Meng-Qi Han, Yan Liu, Kuo-Fang Chung

**Affiliations:** 1 Guangxi Key Laboratory of Plant Conservation and Restoration Ecology in Karst Terrain, Guangxi Institute of Botany, Guangxi Zhuangzu Autonomous Region and Chinese Academy of Sciences, Guilin, 541006 China; 2 School of Life Sciences, Fudan University, Shanghai, 200433 China; 3 College of Life Sciences, Guangxi Normal University, Guilin, 541004 China; 4 Research Museum and Herbarium (HAST), Biodiversity Research Center, Academia Sinica, Taipei, 11529 Taiwan

**Keywords:** Limestone flora, Loxocarpinae, *Paraboea dushanensis*, *Paraboea sinovietnamica*, *Paraboea xiangguiensis*, Sino-Vietnamese limestone karsts (SVLK)

## Abstract

**Background:**

The limestone karsts of Southeast Asia and South China are a major biodiversity hotspot of global terrestrial biomes. With more than 130 described species, *Paraboea* has become one of the most characteristic plant groups in the Southeast Asian limestone flora. During the course of extensive field work on the limestone formations of southern and southwestern China, three unknown species of *Paraboea* were collected.

**Results:**

Molecular phylogenetic analyses based on nuclear ITS and chloroplast *trnL*-*F* sequences strongly confirm the placements of the three new species in *Paraboea* sensu Puglisi et al. (Taxon 65:277–292. 10.12705/652.5, 2016). Moreover, these three novelties can be distinguished from known *Paraboea* species with distinct morphological characters, further supporting their recognition as new species.

**Conclusions:**

With the support of detailed morphological studies and molecular phylogenetic analyses, *Paraboea dushanensis*, *P. sinovietnamica* and *P. xiangguiensis* are recognized as three species new to science.

**Electronic supplementary material:**

The online version of this article (10.1186/s40529-017-0207-5) contains supplementary material, which is available to authorized users.

## Background

As currently circumscribed, the Asian gesneriad genus *Paraboea* (C.B.Clarke) Ridl. comprises ca. 130 species of rosulate or caulescent herbs characterized by the abaxially matted leaves with densely interwoven indumentum and flowers with flat-faced to shortly campanulate corolla and non-erect anthers (Middleton et al. 2010; Puglisi et al. 2016). A majority of *Paraboea* species are lithophytes on limestone substrates, distributed in South China, northeastern India and the eastern Himalayas, Indochina, and Malesia as far east as Sulawesi (Middleton et al. 2010). Since the last major revision by Xu et al. (2008) in which 89 species and 5 varieties were recognized, *Paraboea* has been expanded to include the ca. 20 species of *Phylloboea* Benth. and *Trisepalum* C.B.Clarke (Puglisi et al. 2011), with the reduction of five taxa constituting the new genus *Middletonia* C.Puglisi (Puglisi et al. 2016). More than 30 new species have also been described since the revision by Xu et al. (2008), almost all narrowly distributed endemic from limestone karsts (Chen et al. 2008, 2012; Kiew 2010; Triboun and Middleton 2012, 2015; Xu et al. 2012a; Triboun 2013; Wen et al. 2013; Puglisi et al. 2015; Guo et al. 2016; Wen and Wei 2016). Because a great proportion of Asian limestone karsts remain unexplored or underexplored, it is fully expected that additional new species of *Paraboea* will be unearthed given that further field investigations and herbarium work are conducted (Puglisi et al. 2015).

Sino-Vietnamese limestone karsts (SVLK) are vast terrains striding across the border between China and Vietnam (Xu et al. 2012b; Chung et al. 2014), renowned for their spectacular landscape and rich biodiversity (Myers et al. 2000; Clements et al. 2006; Hou et al. 2010; López-Pujol et al. 2011). Botanically, the SVLK are home to a myriad of species-rich genera with narrowly endemic entities (Chung et al. 2014) such as *Aspidistra* Ker-Gawler (e.g., Liu et al. 2011, 2016), *Begonia* L. (e.g., Peng et al. 2014, 2015), *Elatostema* Forster & Forster (e.g., Wei et al. 2011), *Impatiens* L. (e.g., Tan et al. 2015; Yu et al. 2015), *Polystichum* Roth (He and Zhang 2011; Zhang and He 2011), and several genera of Gesneriaceae (Wei 2010; Xu et al. 2012b, 2014; Guo et al. 2015) including *Paraboea* (Xu et al. 2012a; Guo et al. 2016).

During the course of extensive floristic surveys in limestone karsts of southern and southwestern China in recent years, we collected three species of *Paraboea* with spectacular flowers and/or fruits not known previously. After consulting the relevant literature (Burtt 1984; Wang et al. 1990, 1998; Li and Wang 2004; Chen et al. 2008, 2012; Xu et al. 2008, 2012a; Kiew 2010; Triboun and Middleton 2012; Wen et al. 2013; Guo et al. 2016; Wen and Wei 2016), as well as herbarium specimens of E, GXMI, HITBC, IBK, IBSC, KUN, and PE (herbarium acronyms according to Index Herbariorum; Thiers 2017), they were identified as three new species of *Paraboea* based on detailed examination of salient morphological and anatomical features and molecular phylogenetic analyses.

## Methods

### Taxon sampling and DNA sequencing

For phylogenetic analyses, a majority of species of *Paraboea* available in GenBank were used, with nine additional species endemic to China sampled. A total of 83 accessions representing 67 species of *Paraboea* were included in this study. Based on Puglisi et al. (2016), two species of *Ornithoboea* Parish ex C.B.Clarke and three species of *Middletonia* C.Puglisi were chosen as outgroups. Species, voucher information, and NCBI accession numbers are listed in Additional file 1. Two molecular markers, including the nuclear ITS (internal transcribed spacer) and the chloroplast *trnL*-*F* intron-spacer region (*trnL*-*F*), were used in this study. Total genomic DNA was extracted from silica gel-dried leaf materials using the CTAB protocol (Doyle and Doyle 1987). The primers ITS-4 and ITS-5 (Möller and Cronk 1997) were used to amplify and sequence the ITS region. The primers *trnL*-*F e* and *f* (Taberlet et al. 1991) were used to amplify and sequence the cp DNA region based on the PCR procedures outlined in Guo et al. (2016). The PCR products were purified using the Tian quick Midi Purification Kit (TianGen Biotech, Beijing, China) and directly sequenced. Sequencing reactions were performed using the ABI Prism Bigdye Terminator Cycle Sequencing Kit (Applied Biosystems, Foster City, California, USA). Sequences were analyzed using an ABI 3730 DNA Sequencer. The program Sequencher 5.0 (Gene Codes Co., Ann Arbor, Michigan, USA) was used to evaluate chromatograms for base confirmation and to edit contiguous sequences. Sequences were initially aligned using MUSCLE 3.8.31 (Edgar 2004), followed by manual adjustments in Geneious 9.1.2 (http://www.geneious.com, Kearse et al. 2012).

### Phylogenetic analyses

The phylogenetic analyses were conducted based on maximum likelihood (ML) and Bayesian inference (BI) methods for the individual locus datasets (ITS/*trnL*-*F*) and combined dataset (ITS-*trnL*-*F*), using RAxML v7.0.4 (Stamatakis et al. 2008) and MrBayes v3.3.5 (Ronquist et al. 2012), respectively. The model GTR + Ґ was selected as the optimal model for both DNA regions based on the Akaike Information Criterion via jModeltest v2.1.4 (Posada 2008). For ML analyses, node support was estimated with nonparametric bootstrap (1000 replicates) following a thorough search for the best ML tree. For BI analyses, four runs of Metropolis-coupled Markov chain Monte Carlo (MCMCMC) analyses were conducted with one tree sampled for every 2000 generations over 20 million generations, starting with a random tree. Analyses were run until the average standard deviation of the split frequencies approached 0.01, indicating that two runs converged to a stationary distribution. The first 25% of sampled trees corresponding to the burn-in period was discarded, and the remaining trees were used to construct a majority-rule consensus tree. We used bootstrap support (BS) ≥ 70% and posterior probability (PP) ≥ 0.95 as the thresholds for strongly supported clades (Wang et al. 2014). To investigate congruence between the nuclear and chloroplast genomes, topologies of the ITS and *trnL*-*F* datasets of both ML and BI analyses were compared. Because a majority of clades with BS ≥ 70% and PP ≥ 0.95 were congruent without significant conflicts, the concatenated dataset was presented for further discussion.

## Results and discussion

**Fig. 1:**
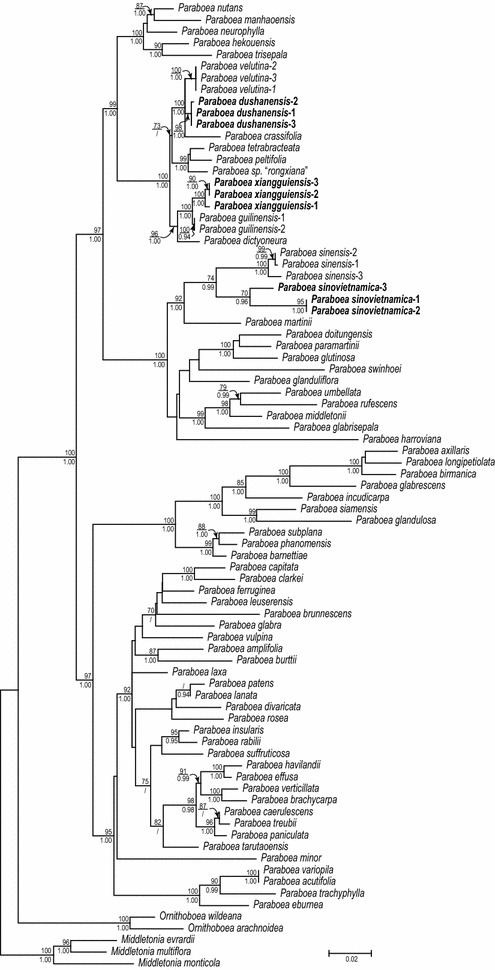
The best ML tree from the analyses of the combined ITS and chloroplast *trnL*-*F* regions. ML bootstrap support values (> 70%) and Bayesian posterior probability > 0.90 are shown above and below the branch around the corresponding node. The three new species are highlighted in bold

The concatenated DNA matrix (78 ITS sequences and 81 *trnL*-*F* sequences) had a length of 1780 aligned characters (ITS: 812 bp, *trnL*-*F*: 968 bp), of which 645 (ITS: 454 bp, *trnL*-*F*: 191 bp) are variable and 415 (ITS: 332 bp, *trnL*-*F*: 83 bp) are parsimony informative. The best ML phylogram with bootstrap (BS) supports and posterior probability (PP) values of Bayesian analyses is depicted in Fig. 1. The phylogenetic relationships of the concatenated matrix are congruent with those reported in Puglisi et al. (2011, 2016). Samples of the three new species (*Paraboea dushanensis*, *P. sinovietnamica* and *P. xiangguiensis*) are shown as distinct clades grouped within *Paraboea* sensu Puglisi et al. (2016) with strongest support values (BS = 100%, PP = 1.00), ascertaining their recognition as distinct species of *Paraboea*. Multiple samples identified as *Paraboea dushanensis*, *P. xiangguiensis*, and *P. sinovietnamica* are all monophyletic (BS = 98, 100, 70%, PP = 1.00, 1.00, 0.96), and each of these three new species is placed as sister group of its morphologically most similar congener [i.e., *P. velutina* (W.T.Wang & C.Z.Gao) B.L.Burtt, *Paraboea crassifolia* (Hemsl.) B.L.Burtt, *P. guilinensis* L.Xu & Y.G.Wei, and *P. sinensis* (Oliv.) B.L.Burtt] with strong support (Fig. 1).

## Conclusions

### Taxonomic treatment

***Paraboea dushanensis*** W.B.Xu & M.Q.Han, **sp. nov.**

独山蛛毛苣苔 (Figs. 2, 3)

**Fig. 2:**
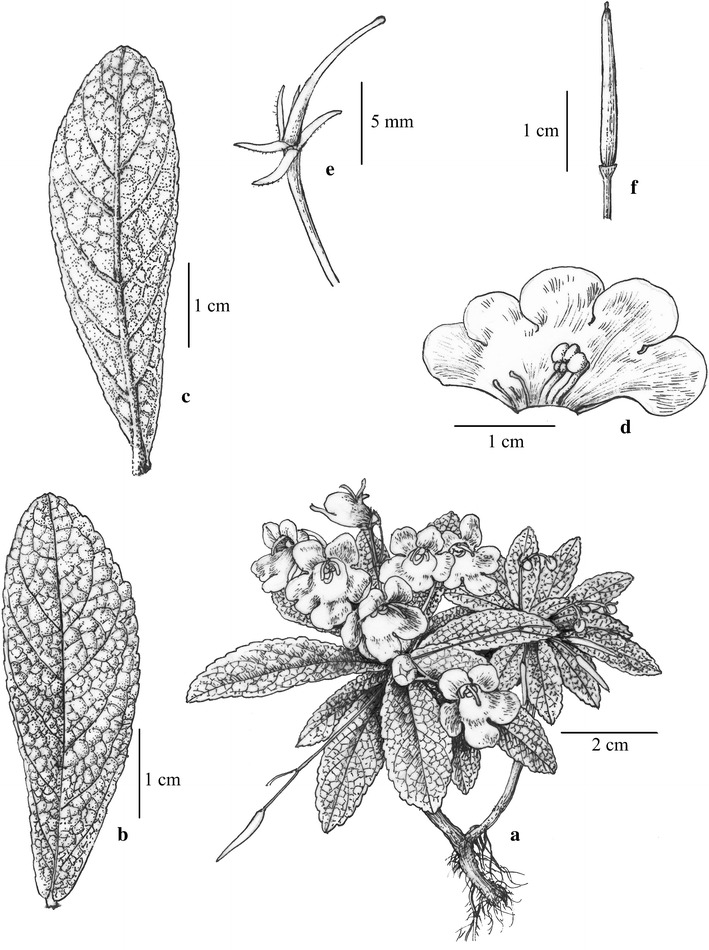
*Paraboea dushanensis* W.B.Xu & M.Q.Han. **a** Habit; **b** upper surface of leaf; **c** lower surface of leaf; **d** opened corolla showing stamens and staminodes. **e** Pistil and calyx; **f** capsule (Drawn by W.H. Lin from the holotype)

**Fig. 3:**
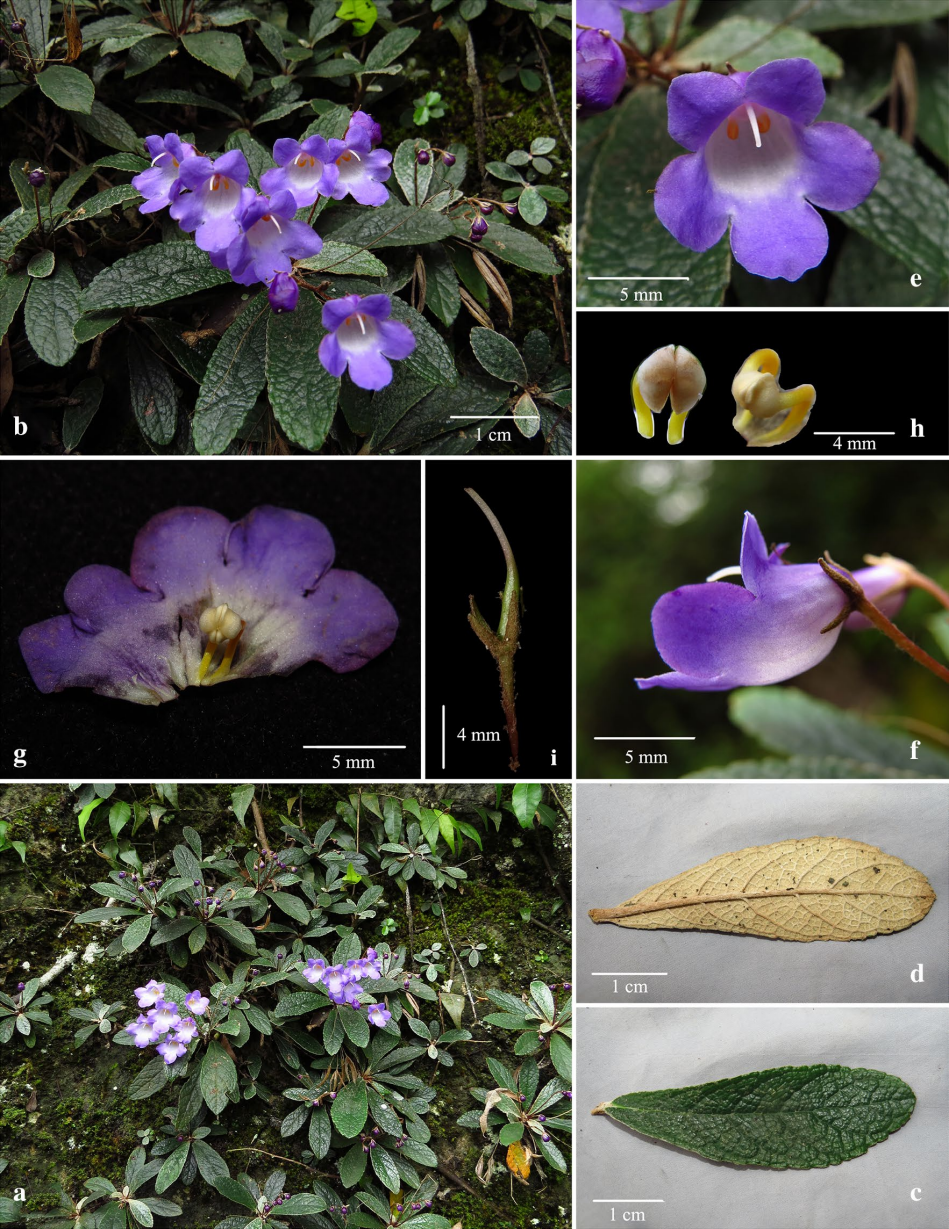
*Paraboea dushanensis* W.B.Xu & M.Q.Han. **a** Habitat; **b** habit; **c** upper surface of leaf; **d** lower surface of leaf; **e** flower face view; **f** flower side view; **g** opened corolla showing stamens and staminodes; **h** stamens; **i** pistil and calyx

**Fig. 4:**
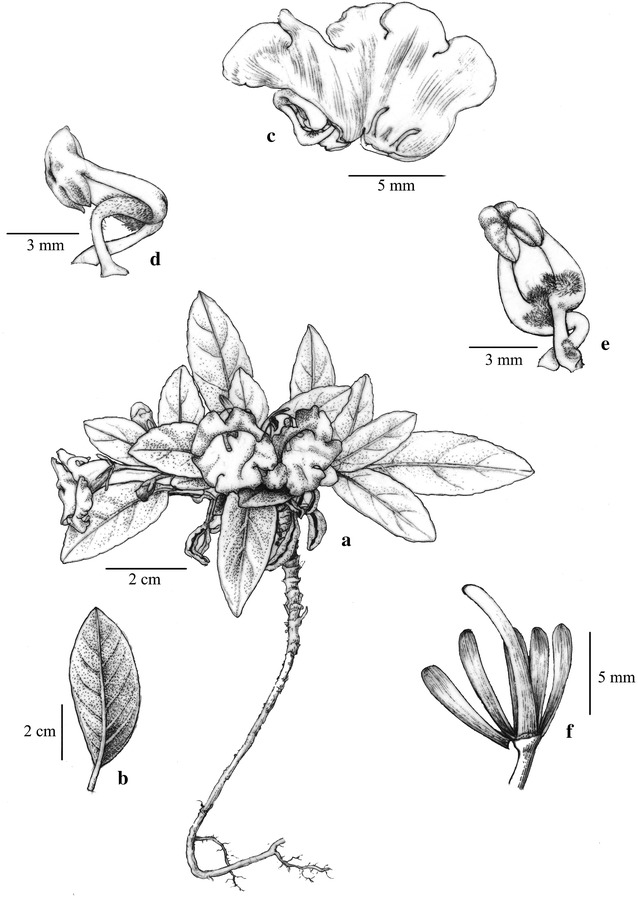
*Paraboea sinovietnamica* W.B.Xu & J.Guo. **a** Habit; **b** lower surface of leaf; **c** opened corolla showing stamens and staminodes; **d** stamens side view; **e** stamens face view; **f** pistil and calyx (Drawn by W.H. Lin from the holotype)

**Fig. 5:**
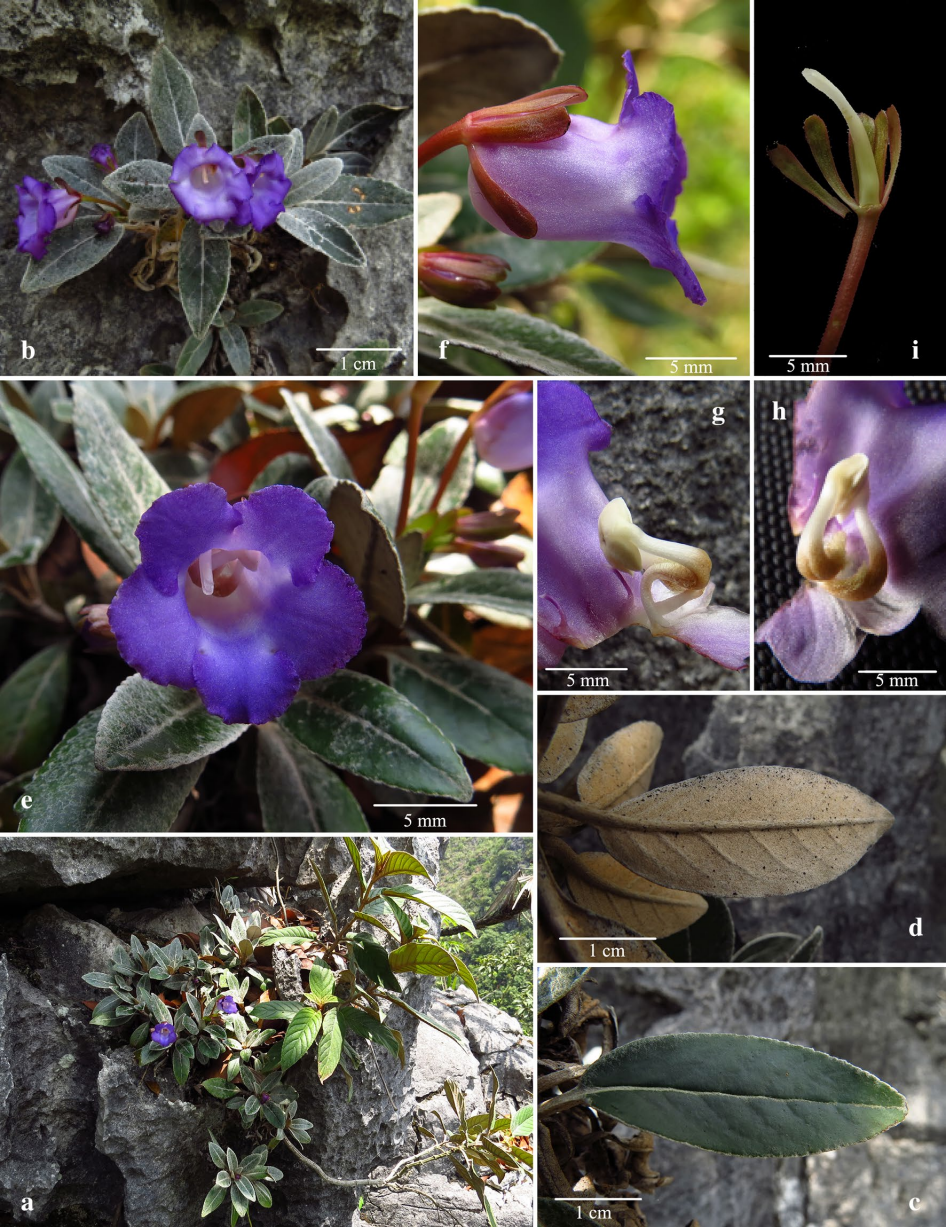
*Paraboea sinovietnamica* W.B.Xu & J.Guo. **a** Habitat; **b** habit; **c** upper surface of leaf; **d** lower surface of leaf; **e** flowers face view; **f** flowers side view; **g** stamens side view; **h** stamens face view; **i** pistil and calyx

**Fig. 6:**
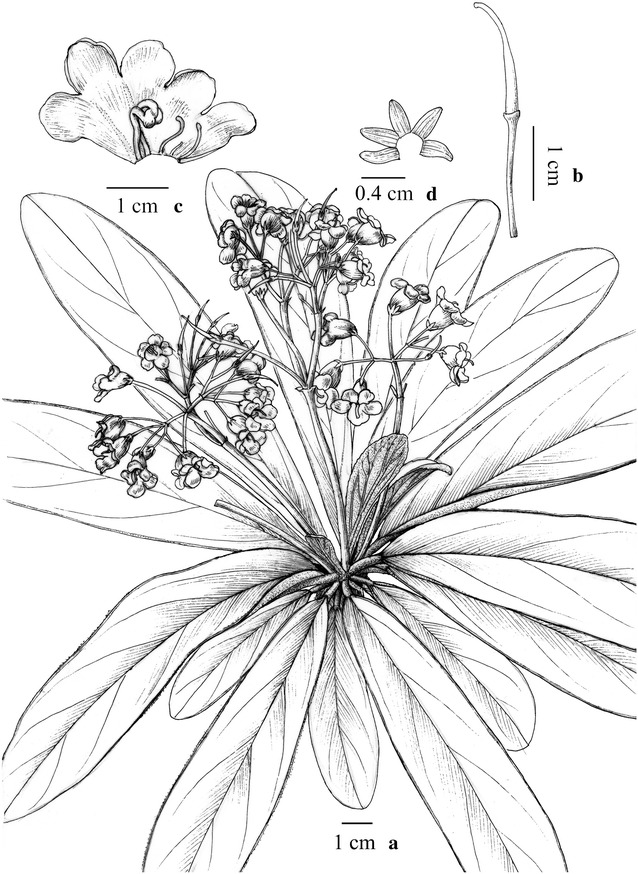
*Paraboea xiangguiensis* W.B.Xu & B.Pan. **a** Habit; **b** pistil; **c** opened corolla showing stamens and staminodes; **d** calyx (Drawn by W.H. Lin from the holotype)

**Fig. 7:**
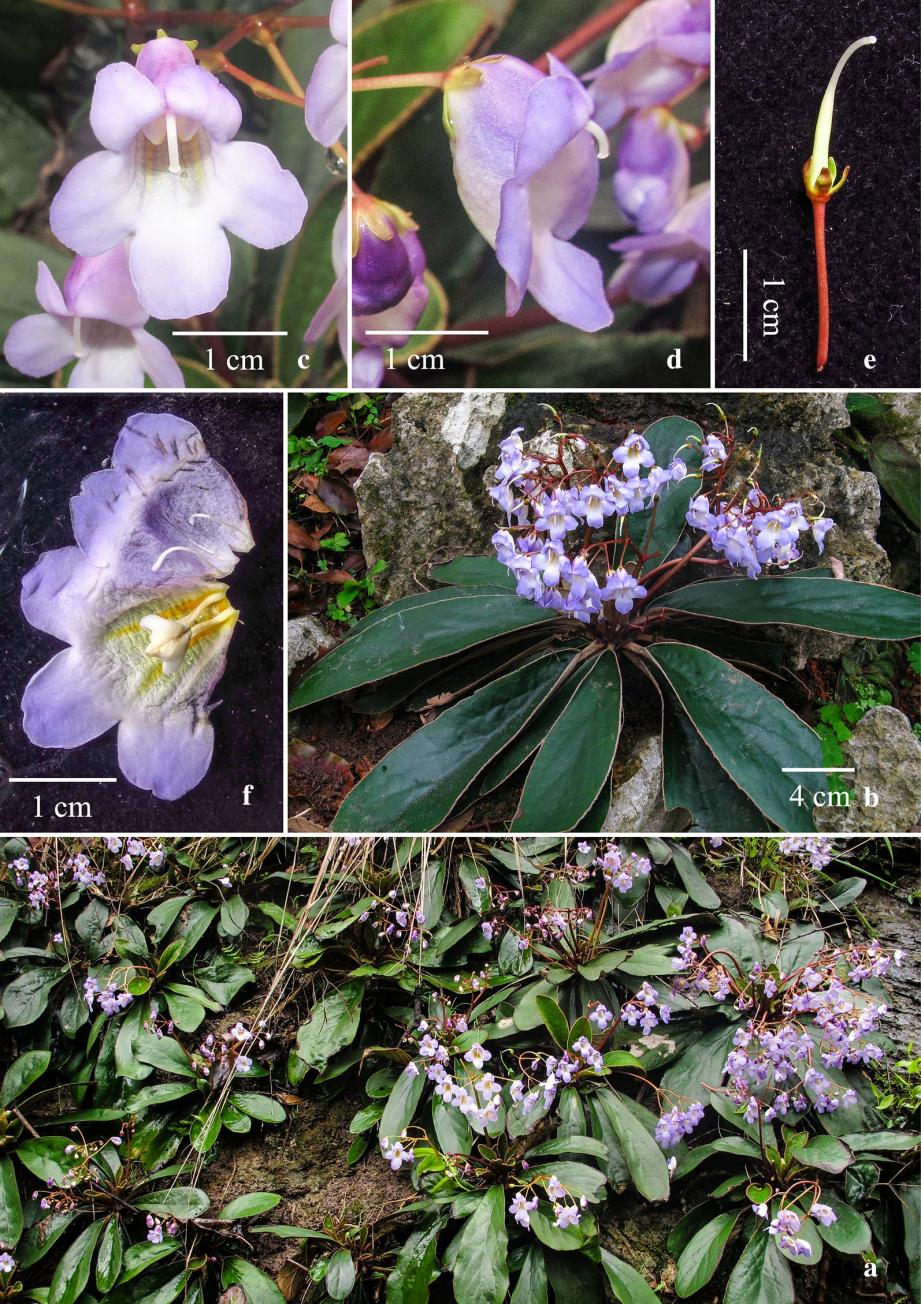
*Paraboea xiangguiensis* W.B.Xu & B.Pan. **a** Habitat; **b** habit; **c** flowers face view; **d** flowers side view; **e** pistil and calyx; **f** opened corolla showing stamens and staminodes

Diagnosis: *Paraboea dushanensis* is similar to *P. velutina* (W.T.Wang & C.Z.Gao) B.L.Burtt, but it can be distinguished by having 6–13 leaves, congested at the apex of the rhizome (vs. many leaves spirally arranged, crowded near branch apex), the large leaf blade 4–8 × 0.7–1.5 cm (vs. 0.9–2.3 × 0.4–1.0 cm), the calyx lobes 3.5–5 × 1.0–1.2 mm, with ferrugineous matted indumentum outside (vs. 1.0–1.2 × 0.3 mm, outside glandular-puberulent), the corolla 1.1–1.6 cm long, purple–blue, outside glabrous (vs. 4–5 cm long, white, outside glandular-puberulent).

Type: CHINA. Guizhou: Dushan County, Xiasi Town, 920 m, 25°27′N, 107°30′E, 26 May 2015, *Wei*-*Bin Xu* et al*. 12319* (holotype IBK, isotypes PE, HAST).

Perennial herbs: Rhizomes subterete, 4–10 cm long, 2–6 mm in diameter, branched at the apex of rhizome. Leaves 6–13, congested at the apex of rhizome, subsessile, petiole 1.5–3.5 mm long, ca. 1.5 mm in diameter, covered with grayish to brownish matted indumentum, leaf blade 4–8 × 0.7–1.5 cm, 3–6 times as long as wide, oblanceolate, rarely oblong, leathery, bases cuneate to attenuate, margins crenate to shallowly repand, apices obtuse to subround, upper leaf surfaces covered with arachnoid indumentum when young, but glabrescent at maturity, lower leaf surfaces with ferrugineous matted indumentum, lateral veins 5–8 on each side of midrib, impressed adaxially and prominent abaxially, tertiary venation conspicuously reticulate on the upper and lower leaf surface. Inflorescences cymose, axillary, 1 (rarely 2)-branched, 1–5-flowered; peduncles 3–5 cm long, 0.8–1 mm in diameter, covered with ferrugineous matted indumentum; bracts 2, opposite, 3–5 × 1–1.5 mm, linear-lanceolate, margins entire, apices acute, with ferrugineous matted indumentum outside and glabrous inside; pedicels 0.6–1.5 cm long, ca. 0.7 mm in diameter, covered with ferrugineous matted indumentum. Calyx 3.5–5 mm long, 5-parted nearly to the base, lobes linear-lanceolate, 1.0–1.2 mm wide, with ferrugineous matted indumentum outside and glabrous inside, margins entire. Corolla 1.1–1.6 cm long, purple–blue, outside and inside glabrous; tube 6–9 mm long, 5–8 mm in diameter at the mouth; the limb distinctly 2-lipped, adaxial lip 2-lobed to near base, lobes broadly ovate, 4–5 × ca. 4 mm, abaxial lip 3-lobed to over middle, lobes oblong, 5–6 × 4–5 mm. Stamens 2, adnate to the corolla base; filaments 4–6 mm long, yellow, glabrous; anthers elliptic, ca. 2.5 mm long; staminodes 3, glabrous, lateral ones 2.5–3 mm long, adnate to the corolla tube base; middle one 2–2.5 mm long, adnate to the corolla tube base. Pistil glabrous; ovary 5–6.5 mm long, ca. 1.5 mm in diameter, style 6–9 mm long, stigma slightly capitate. Capsule not twisted, 1.2–3.1 cm long, ca. 3 mm in diameter, glabrous.

**Fig. 8:**
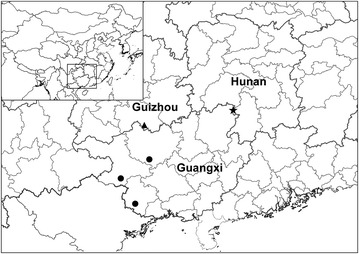
Distribution of *Paraboea dushanensis* W.B.Xu & M.Q.Han (triangle), *Paraboea sinovietnamica* W.B.Xu & J.Guo (circle), and *Paraboea xiangguiensis* W.B.Xu & B.Pan (star) in China

Distribution, habitat and ecology: *Paraboea dushanensis* is only found at the type locality on limestone substrate (Fig. 8), and only one population has so far been identified by us during field investigations in 2015. *Paraboea dushanensis* grows on rock faces of the limestone karst, at an elevation between 900 and 960 m.

Phenology: This new species had been observed in flower from May to June, and fruit from July to August.

Etymology: The specific epithet is derived from the type locality, Dushan County, Southern Guizhou Province.

**Fig. 9:**
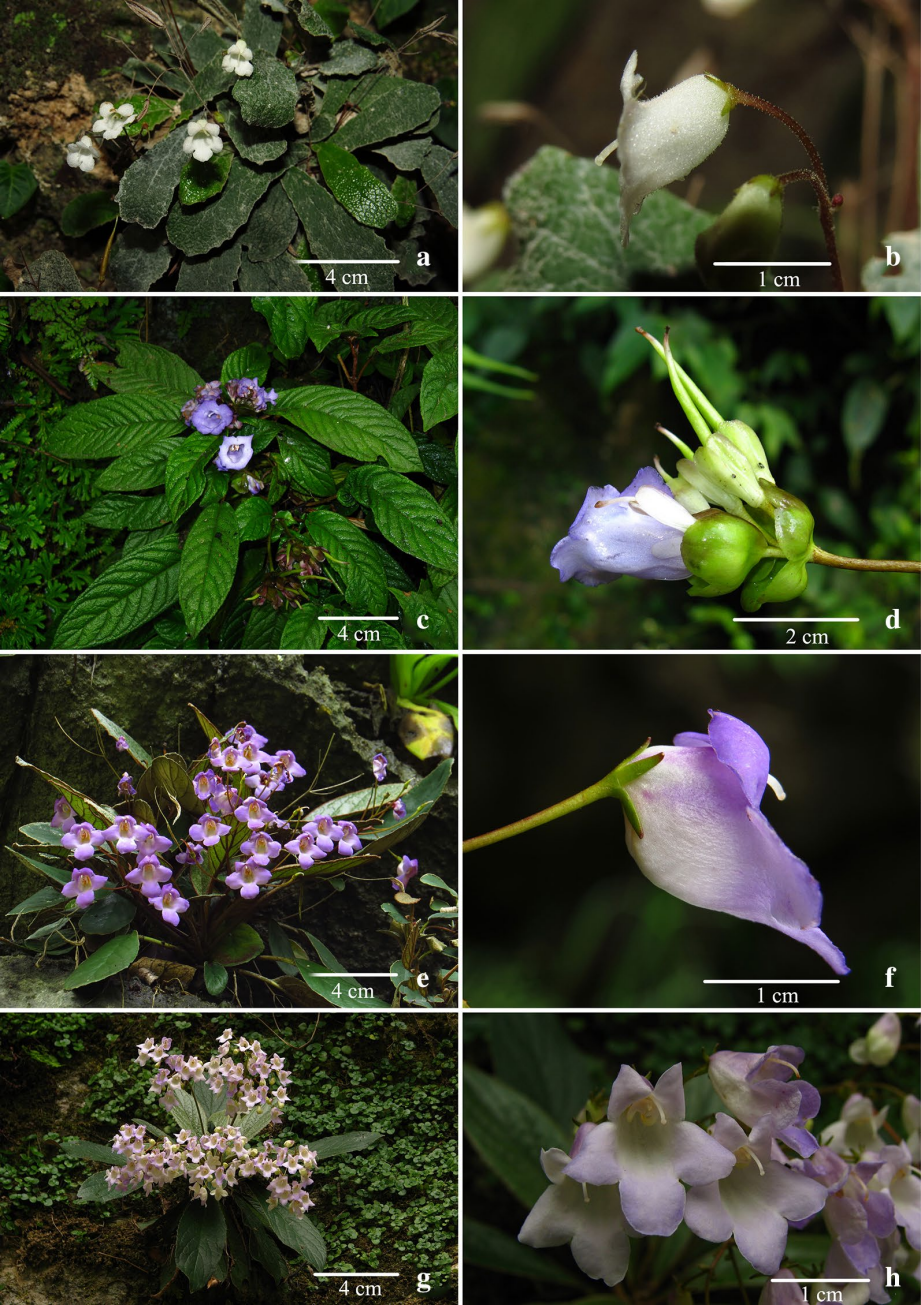
*Paraboea velutina* (W.T.Wang & C.Z.Gao) B.L.Burtt. **a** Habit; **b** flower side view. *Paraboea sinensis* (Oliv.) B.L.Burtt. **c** Habit; **d** flower side view. *Paraboea guilinensis* L.Xu & Y.G.Wei. **e** Habit; **f** flower side view. *Paraboea dictyoneura* (Hance) B.L.Burtt. **g** Habit; **h** flowers face and side view

Notes: *Paraboea dushanensis* is most similar to *P. velutina* (Fig. 9a, b) in the habit and the leaf blade shape, but it can be distinguished from the latter by many leaf characters (see Diagnosis). *Paraboea dushanensis* is also similar to *Paraboea crassifolia* (Hemsl.) B.L.Burtt, but differs in the obvious subterete rhizomes, the branching at the apex of rhizome (vs. rhizomes very unobvious, rosulate), the leaf blade oblanceolate, rarely oblong, 4–8 × 0.7–1.5 cm, 3–6 times as long as wide, (vs. obovate, 3–16 × 1.5–5 cm, 2–3 times as long as wide), tertiary venation conspicuously reticulate on the upper leaf surface (vs. smooth on the upper leaf surface), calyx 3.5–5 mm long (vs. 2 mm long), capsule not twisted (vs. spirally twisted). Phylogenetic analyses revealed that these three species are closely related and yet considerably different from each other, supporting the recognition of *P. dushanensis* as a new species.

Additional specimens examined (paratypes): CHINA. Guizhou: Dushan County, Xiasi Town. 920 m, 26 May 2015, *Wei*-*Bin Xu* et al*. 12320* (IBK); ibid., *Wei*-*Bin Xu* et al. *12321* (IBK), 17 May 2015, *Meng*-*Qi Han & Jin*-*Quan Huang HMQ318* (IBK).

***Paraboea sinovietnamica*** W.B.Xu & J.Guo, **sp. nov.**

中越蛛毛苣苔 (Figs. 4, 5)

Diagnosis: *Paraboea sinovietnamica* resembles *P. sinensis* (Oliv.) B.L.Burtt in the corolla shape, but differs in being a smaller herb (vs. herb to subshrub, stem to 1.5 m high), the leaf blade thickly papery to thinly leathery, 2.5–6 × 0.8–2.0 cm (vs. papery, 5.5–26 × 2–10 cm), the cymes, 1–3-flowered (vs. 10 to more flowered), the peduncle 1–3 cm long, glabrous (vs. 2.5–6 cm long, brown-pannose), the bracts oblong to oblong-spathulate, 4–5 × 2–3 mm, (vs. suborbicular to broadly ovate, 10–15 × 8–12 mm), the filaments bearded at the middle (vs. pubescent to glabrous).

Type: CHINA. Guangxi: Jingxi County, Wuping Town. 1000 m, 23°12′39″N, 106°30′56″E, 1 June 2015, *Wei*-*Bin Xu* et al. *12366* (holotype IBK, isotypes HAST, PE).

Perennial herbs: Rhizomes subterete, 4.5–16 cm long, 2–4 mm in diameter, erect stem short and internodes inconspicuous. Leaves 6–16, congested at the apex of the rhizome, petiole 0.4–2.6 cm long, leaf blade 2.5–6 × 0.8–2.0 cm, 2–3 times as long as wide, elliptic to long-elliptic, thickly papery to thinly leathery, bases cuneate, inequilateral, margins entire, apices obtuse, upper leaf surfaces covered with arachnoid when young, but glabrescent in age, lower leaf surfaces with grayish to brownish matted indumentum, lateral veins 5–7 on each side of midrib, smooth adaxially and prominent abaxially. Inflorescences cymose, axillary, 0–1-branched, 1–3-flowered; peduncles 1–3 cm long, ca. 1.5 mm in diameter, glabrous; bracts 2, opposite, 4–5 × 2–3 mm, oblong to oblong-spathulate, margins entire, apices obtuse, outside and inside glabrous; pedicels 0.6–1.7 cm long, ca. 1 mm in diameter, glabrous. Calyx 5–9 mm long, 5-parted nearly to the base, lobes oblong-spathulate, 1.1–1.5 mm wide, outside and inside glabrous, margins entire, apices round. Corolla 1.5–2.3 cm long, purple–blue, outside and inside glabrous; tube 8–14 mm long, 8–12 mm in diameter at the mouth; the limb inconspicuously 2-lipped, adaxial lip 2-lobed to near base, lobes round, 5–7 × ca. 6 mm, abaxial lip 3-lobed to over middle, lobes round, 6–8 × ca. 6 mm. Stamens 2, adnate to the corolla base; filaments 9–12 mm long, doubly geniculate, bearded at the middle; anthers elliptic, ca. 4.0 mm long; staminodes 3, glabrous, lateral ones 2–3 mm long, adnate to the corolla tube base, apex expanded; middle one ca. 1.5 mm long, adnate to the corolla tube base. Pistil glabrous; ovary 4–7 mm long, ca. 1.4 mm in diameter, style 5–8 mm long, stigma not capitate. Capsule spirally twisted, 3.0–6.5 cm long, ca. 2–3 mm in diameter, glabrous.

Distribution, habitat and ecology: *Paraboea sinovietnamica* is found near the border between China and Vietnam on limestone substrate, and only three populations have been identified so far by us during field investigations from 2006 to 2015 (Fig. 8). *Paraboea sinovietnamica* grows on rock faces of the limestone karst top, at an elevation between 400 and 1000 m.

Phenology: This new species had been observed in flower from May to June, and fruit from July to August.

Etymology: The specific epithet is derived from the type locality, the Sino-Vietnamese border.

Notes: *Paraboea sinovietnamica* is most similar to *P. sinensis* (Fig. 9c, d), but it is easily distinguished from the latter by the habit, leaves, inflorescences, and flowers (see Diagnosis). Phylogenetic analyses revealed that these two species are closely related and yet considerably different from one another, supporting the recognition of *P. sinovietnamica* as a new species.

Additional specimens examined (paratypes): CHINA. Guangxi: Jingxi County, Wuping Town, 1000 m, 1 June 2015, *Wei*-*Bin Xu* et al. *12365* (IBK); ibid., 16 June 2010, *Wei*-*Bin Xu et Yu*-*Song Huang. 10656* (IBK); ibid., 31 May 2006, *Hai*-*Ning Qin* et al. *531031* (PE & IBK); ibid., 5 June 2011, *Yu*-*Song Huang & Dong*-*Xin Nong Y0624* (IBK); Ningming County, Nonggang National Nature reserve, Longrui, 430 m, 5 May 2009, *Yu*-*Song Huang H09388* (IBK); ibid., 25 May 2008, *Joint Expedition on Plants in Guangxi of CAS 1187* (IBK, KUN, PE); ibid., 14 September 2003, *Wai*-*Chao Leong* et al. 3642 (HAST); Dahua County, Yalong Twon, 800 m, 29 April 2015, *Wei*-*Bin Xu* et al. *12217* (IBK).

***Paraboea xiangguiensis*** W.B.Xu & B.Pan, **sp. nov.**

湘桂蛛毛苣苔 (Figs. 6, 7)

Diagnosis: *Paraboea xiangguiensis* resembles *P. guilinensis* L.Xu & Y.G.Wei in the corolla shape, but differs in the rhizomes inconspicuous, 1.5–2 cm long, simple at the apex (vs. short caulis or conspicuous rhizomes, 2–13 cm long, branched or simple at the apex), the leaves subsessile or with a very short petiole up to 2 cm long (vs. obviously petiolate, petiole 1.4–7.0 cm long), the leaf blade spathulate, narrowly obovate to obovate-elliptic, 9–20 × 2.5–4.6 cm, base attenuate (vs. obovate-elliptic or elliptic, 2.8–5.8 × 1.5–2.2 cm, base rounded to broadly cuneate), the cymes, 2–3-branched, 6–22-flowered (vs. 1–2-branched, 3–8-flowered).

Type: CHINA. Guangxi: Quanzhou County, Huangshahe Town. 240 m, 26°03′N, 111°13′E, 20 March 2013, *Wei*-*Bin Xu* & *Bo Pan 11918* (holotype IBK, isotypes HAST, PE).

Perennial rosulate herbs: Rhizomes subterete, 1.5–2 cm long, ca. 10 mm in diameter. Leaves 8–16, congested at the apex of the rhizome, subsessile or with a short petiole up to 2 cm long, leaf blade 9–20 × 2.5–4.6 cm, 3.5–4.5 times as long as wide, spathulate, narrowly obovate to obovate-elliptic, thickly leather, bases attenuate, margins entire to shallowly repand, apices obtuse to subround, upper leaf surfaces covered with arachnoid indumentum when young, but glabrescent at maturity, lower leaf surfaces with ferrugineous matted indumentum, lateral veins 5–7 on each side of midrib, smooth adaxially and prominent abaxially. Inflorescences cymose, axillary, 2–3-branched, 6–22-flowered; peduncles 7–13 cm long, 2–3 mm in diameter, glabrous; bracts 2, opposite, 2–3 × ca. 1.5 mm, lanceolate, margins entire, apices acute, outside and inside glabrous; pedicels 0.8–2.7 cm long, ca. 0.6 mm in diameter, glabrous. Calyx 3–4 mm long, 5-parted nearly to the base, lobes lanceolate, 1.2–1.5 mm wide, outside and inside glabrous, margins entire. Corolla 1.2–2.1 cm long, purplish, outside and inside glabrous; tube 8–12 mm long, 8–11 mm in diameter at the mouth; the limb distinctly 2-lipped, adaxial lip 2-lobed to near base, lobes broadly ovate, 5–6 × ca. 6 mm, abaxial lip 3-lobed to over middle, lobes broadly ovate, 6–7 × ca. 6 mm. Stamens 2, adnate to the corolla base; filaments 9–11 mm long, glabrous; anthers elliptic, ca. 4.0 mm long; staminodes 3, glabrous, lateral ones 6–8 mm long, adnate to the corolla tube base, apex expanded; middle one ca. 5 mm long, adnate to the corolla tube base. Pistil glabrous; ovary 6–8 mm long, ca. 1.5 mm in diameter, style 6–7 mm long, stigma capitate. Dehisced fruit a slightly twisted capsule, 3.4–5.0 cm long, ca. 2–3 mm in diameter, glabrous.

Distribution, habitat and ecology: *Paraboea xiangguiensis* is only found along the border between Hunan Province and Guangxi Zhuangzu Autonomous Region on limestone substrate (Fig. 8), and only four populations have so far been identified by us during field investigations in 2013 and 2016. *Paraboea xiangguiensis* grows on moist rock faces of the limestone karst, at an elevation between 200 and 250 m.

Phenology: This new species had been observed in flower from March to April.

Etymology: The specific epithet is derived from the type locality, the border between Hunan province (Abbr. Xiang) and Guangxi Zhuangzu Autonomous Region (Abbr. Gui).

Notes: *Paraboea xiangguiensis* is most similar to *P. guilinensis* (Fig. 9e, f) in its corolla shape, but it is easily distinguished from the latter by habit, leaves, and inflorescences (see Diagnosis). *Paraboea xiangguiensis* is also similar to *P. dictyoneura* (Fig. 9g, h), differing in leaf shape (blade spathulate, narrowly obovate to obovate-elliptic vs. narrowly obovate-elliptic), size (9–20 × 2.5–4.6 vs. 7–15 × 2–4.5 cm), margins (entire to shallowly repand vs. more or less serrullate), apex (obtuse to subround vs. acute to obtuse), and hairiness of peduncle and pedicel (glabrous vs. grayish matted indumentum) and bract and calyx (glabrous outside vs. grayish matted indumentum). Phylogenetic analyses revealed that these three species are closely related and yet considerably differentiated from one another, supporting the recognition of *P. xiangguiensis* as a new species.

Additional specimens examined (paratypes): CHINA. Guangxi: Quanzhou County, Huangshahe Town. 220 m, 7 July 2016, *Wei*-*Bin Xu* & *Jing Guo 13006* (IBK); ibid., 7 July 2016, *Wei*-*Bin Xu* & *Jing Guo 13007* (IBK); ibid., 7 July 2016, *Wei*-*Bin Xu* & *Jing Guo 13008* (IBK); ibid., 20 March 2013, *Bo Pan & Chun*-*Rui Lin H2159* (IBK); ibid., 20 March 2013, *Quanzhou Exped. 450324130320003LY* (IBK). Hunan: Yongzhou City, Shiyantou Town, 250 m, 20 March 2013, *Bo Pan* et al. *Y2159* (IBK).

## Additional file

**Additional file 1: Appendix S1.** *Taxon*: NCBI accession numbers (ITS/*trnL-F*), and voucher information.
