# Supplementary material for: Three new species of Paraboea (Gesneriaceae) from limestone karsts of China based on morphological and molecular evidence
Source: Bot Stud. 2017 Dec 1;58:56. doi: 10.1186/s40529-017-0207-5 (PMC5709257; doi:10.1186/s40529-017-0207-5)
Supplement: Supplementary file 1 — Additional file 1: Appendix S1. Taxon: NCBI accession numbers (ITS/trnL-F), and voucher information. [file 40529_2017_207_MOESM1_ESM.doc]

**Additional file 1: Appendix S1.** *Taxon*: NCBI accession numbers (ITS/*trnL-F*), and voucher information [Geography, date, *Collector number* (herbarium)] (**Taxa and NCBI accession numbers of newly generated DNA sequences in bold**).

*Middletonia evrardii* (Pellegr.) C.Puglisi: KU203790/KU203885, Vietnam, Ninh Thuận PRO., Ninh Hải, 11 xi 2010, *Lý Ngọc Sâm & Phạm Vũ Điệp, Lý 497* (E); *Middletonia monticola* (Triboun & D.J.Middleton) C.Puglisi: KU203789/KU203884, Thailand, Surat Thani, Khlong Phanom National Park, 7 ix 2008, *D.J. Middleton & al., 4363* (BK, BKF, E); *Middletonia multiflora* (R. Br.) C.Puglisi: KU203791/KU203886, Thailand, Sukhothai, Khiri Mat, 12 viii 2012, *D.J. Middleton & al., 5557* (BK, BKF, E); *Ornithoboea arachnoidea* Craib: JN934751/JN934709, Thailand, Chiang Mai, Chiang Dao, 20 ix 2008, *D.J. Middleton & al., 4538* (BK, BKF, E); *Ornithoboea wildeana* Craib: JN934752/JN934710, Thailand, Chiang Mai, Doi Chiang Dao Wildlife Sanctuary, 20 ix 2008, *D.J. Middleton & al., 4531* (BKF, E); *Paraboea acutifolia* (Ridl.) B.L.Burtt: KU203969/-, Thailand, Krabi, Wat Tham Seua, 11 ix 2008, *D.J. Middleton, 4446* (BK, BKF, E); *Paraboea amplifolia* Z.R.Xu & B.L.Burtt: JN934754/JN934712, Thailand, Trang, 30 viii 2009, *P. Triboun, s.n. (EDNA09_02281)*(BK); *Paraboea axillaris* Triboun: KU203848/KU203943, Thailand, Tak, Tah Song Yang District, 10 ix 2009, ex cult. RBGE 20092055, *D.J. Middleton, 4840* (E); *Paraboea barnettiae* C.Puglisi: KU203847/KU203942, Thailand, Peninsular Thailand, *K. Williams & al., 2118* (A); *Paraboea birmanica* (Craib) C.Puglisi: KU203849/KU203944, China, Guangxi, Jingxi, Nan Po, 1 ix 2006, *M. Möller & Y.G. Wei, MMO 06-862b* (E); *Paraboea brachycarpa* (Ridl.) B.L.Burtt: KU203870/KU203965, Malaysia, Pahang, Lipis distr., Gua Bama, *A. Weber, 870508-2/6* (WU); *Paraboea brunnescens* B.L.Burtt: KU203859/KU203954, Thailand, Kanchanaburi, Sisawat, Erawan National Park, 5 viii 2012, *D.J. Middleton & al., 5253* (BK, BKF, E); *Paraboea burttii* Z.R.Xu: KU203858/ KU203953, Thailand, Phatthalung, Khao Banthat Wildlife Sanctuary, Khao Kram Waterfall, 13 ix 2010, *D.J. Middleton, 5407* (BKF, E); *Paraboea caerulescens* (Ridl.) B.L.Burtt: KU203871/KU203966, Malaysia, Perak, Gunung Rapat, *FRIM, FRI 64604* (KEP); *Paraboea capitata* Ridl.: FJ501315/AJ492298, Malaysia, Perak, A. Weber, 870522-5/2 (WU); *Paraboea clarkei* B.L.Burtt: JN934757/JN934715, Malaysia, Sarawak, Bau, Fairy cave, 17 vii 2010, *C.Puglisi, CP 10* (E); *Paraboea crassifolia* (Hemsl.) B.L.Burtt: KU203970/KU204042, China, Guizhou, Jiangkou, 16 ix 2003, *M. Möller & L.M. Gao, MMO 03-322a* (E); *Paraboea dictyoneura* (Hance) B.L.Burtt: KJ475415/FJ501463, China, Guangdong, Liannan, J. Tao and M. Kang LN03 (IBSC)/China, Guangdong, Lianzhou, Xie Qingjian, *J-040 (US422817)* (US); *Paraboea divaricata* (Ridl.) B.L.Burtt: KU203865/KU203960, Thailand, Satun, La Ngu, Mu Ko Phetra National Park, 20 ix 2010, *D.J. Middleton, 5488* (BKF, E); *Paraboea doitungensis* Triboun & D.J.Middleton: KU203846/KU203941, Thailand, Chiang Rai, Mae Fa Luang, Doi Tung, 23 ix 2008, *D.J. Middleton & al., 4576* (BK, BKF, E); ***Paraboea dushanensis* W.B.Xu & M.Q.Han***-*1: **MF358698/MF358714**, China, Guizhou, Dushan, 26 v 2015, *W.B. Xu & J. Guo*, *12319* (IBK); *-*2: **MF358699/ MF358715**, China, Guizhou, Dushan, 26 v 2015, *W.B. Xu & J. Guo*, *12319* (IBK); *-*3: **MF358700/ MF358716**, China, Guizhou, Dushan, 26 v 2015, *W.B. Xu & J. Guo*, *12320* (IBK); Parabo*ea eburnea* Triboun: KU203869/KU203964, Thailand, Ranong, Tham Pha Kayang, 31 vii 2009, *P. Triboun, s.n. (EDNA12_27741)* (BK); *Paraboea effusa* B.L.Burtt: JN934760/JN934718, Malaysia, Sarawak, Mulu, 14 viii 2010, *C.Puglisi, CP 32* (E); *Paraboea ferruginea* (Ridl.) Ridl.: KU203862/-, Malaysia, Kedah, Pulau Langkawi, *A. Weber, 860806* (WU); *Paraboea glabra* (Ridl.) B.L.Burtt: JN934761/JN934719, Thailand, Krabi or Phangnga, *P. Triboun, s.n. (EDNA09_01765)* (BK); *Paraboea glabrescens* (Barnett) C.Puglisi: KU203852/KU203947, Thailand, Kanchanaburi, Thong Pha Phum, 5 viii 2012, *D.J. Middleton & al., 5254* (BK, BKF, E); *Paraboea glabrisepala* B.L.Burtt: JN934762/JN934720, Thailand, Chiang Mai, Doi Chiang Dao Wildlife Sanctuary, 20 ix 2008, *D.J. Middleton & al., 4533* (BK, BKF, E); *Paraboea glanduliflora* Barnett: JN934763/JN934721, Thailand, Chiang Rai, Fang, Doi Ang Khang, 21 ix 2008, *D.J. Middleton & al., 4545* (BK, BKF, E); *Paraboea glandulosa* (B.L.Burtt) C.Puglisi: JN934784/JN934742, Thailand, Kanchanaburi, Thong Pha Phum, 28 x 2009, *D.J. Middleton & P. Triboun, 5202G* (BK, E); *Paraboea glutinosa* (Hand.-Mazz.) K.Y.Pan: JN934764/JN934722, China, Guangxi, Xincheng, 23 viii 2006, *M. Möller & Y.G. Wei, MMO 06-786a* (E); ***Paraboea guilinensis* L.Xu & Y.G.Wei***-*1: **MF358701/MF358717**, China, Guangxi, Linggui, 6 v 2015, *J. Guo*, *166* (IBK); *-*2: KJ475414/KM232657, China, Guangxi, Guilin, *M. Kang & al*, *HXGLZM* (IBSC); *Paraboea harroviana* (Craib) Z.R.Xu: JN934765/JN934723, Thailand, Prachuap Khiri Khan, Khao Loom Muak, 5 ix 2008, *D.J. Middleton & al., 4273* (BK, BKF, E); *Paraboea havilandii* (Ridl.) B.L.Burtt: JN934766/JN934724, Malaysia, Sarawak, Bau, Tai Ton, 21 vii 2010, *C. Puglisi, CP18* (E); *Paraboea hekouensis* Y.M.Shui & W.H.Chen: KU203843/KU203938, China, Yunnan, Hekou, ix 2012, *Shui & al., 94842* (KUN); *Paraboea incudicarpa* B.L.Burtt: JN934767/JN934725, Thailand, Tak, Mae Sot, 11 ix 2009, *D.J. Middleton & P. Triboun, 4857G* (BK, E); *Paraboea insularis* Triboun: KU203857/KU203952, Thailand, Krabi, Ao Luk, *P. Triboun, 3673* (BK); *Paraboea lanata* (Ridl.) B.L.Burtt: -/FJ501467, Malaysia, Kedah, Pulau Langkawi, Pulau Dayang Bunting, *Weber, 860807-1/2* (WU); *Paraboea laxa* Ridl.: -/FJ501466, Malaysia, Kedah, Pulau Langkawi, *C, 4197* (E); *Paraboea leuserensis* B.L.Burtt: KU203863/KU203958, Indonesia, Sumatra, North Sumatra, 9 vii 2011, *C. Puglisi & al., CP 231* (BO, E); *Paraboea longipetiolata* (B.L.Burtt) C.Puglisi: KU203851/KU203946, Thailand, Kanchanaburi, Thong Pha Phum, 6 viii 2012, *D.J. Middleton & al., 5257* (BK, BKF, E); *Paraboea manhaoensis* Y.M.Shui & W.H.Chen: KU203842/KU203937, China, Yunnan, Gejiu, 7 ix 2012, *Shui & al., s.n. (EDNA13_30239)* (KUN); ***Paraboea martinii* (H.Lév.) B.L.Burtt**: **MF358702/MF358718**, China, Yunnan, Luoping, 14 iv 2015 *M.Q. Han & J.Q. Huang*, *HMQ045* (IBK); *Paraboea middletonii* Triboun: KU203845/KU203940, Thailand, Nan, Doi Phu Kha National Park, 15 viii 2012, *D.J. Middleton & al., 5606* (BK, BKF, E); *Paraboea minor* (Barnett) B.L.Burtt: KU203860/KU203955, Thailand, Songkhla, Ton Nga Chang Wildlife Sanctuary, 7 ix 2010, *D.J. Middleton & al., 5225* (BKF, E); *Paraboea neurophylla* (Collett et Hemsl.) B.L.Burtt: JN934769/JN934727, Thailand, Chiang Rai, Mae Fa Luang, 23 ix 2008, *D.J. Middleton & al., 4557* (BK, BKF, E); ***Paraboea nutans* D.Fang & D.H.Qin**: **MF358703/MF358719**, China, Guangxi, Napo, 30 v 2015, *W.B. Xu & J. Guo*, *12345* (IBK); *Paraboea paniculata* (Ridl.) B.L.Burtt: JN934770/JN934728, Malaysia, *FRIM, FRI 65535* (KEP); *Paraboea paramartinii* Z.R.Xu & B.L.Burtt: JN934771/JN934729, China, Guangxi, Napo, 1 ix 2006, *M. Möller & Y.G. Wei, MMO 06-852b* (E); *Paraboea patens* (Ridl.) B.L.Burtt: KU203864/KU203959, Thailand, Phangnga, Phangnga Town Park, 17 ix 2010, *D.J. Middleton & al., 5456* (BKF, E); ***Paraboea peltifolia* D.Fang & L.Zeng**: -/**MF358720**, China, Guangxi, Mashan, 26 v 2015, *W.B. Xu & J. Guo*, *12316* (IBK); *Paraboea phanomensis* Triboun & D.J.Middleton: KU203855/KU203950, Thailand, Surat Thani, Khlong Phanom National Park, 7 ix 2008, *D.J.Middleton & al., 4365* (BK, BKF, E); *Paraboea rabilii* Z.R.Xu & B.L.Burtt: KU203856/KU203951, Thailand, Trang, Huai Yot, *P. Triboun, s.n. (EDNA11_02030)* (BK); *Paraboea rongxiana* F.Wen & Y.G.Wei ined.: KJ475416/KM232659, China, Guangxi, Rongxian, *M. Kang & al*, *GXRX02* (IBSC); *Paraboea rosea* Triboun: KU203866/KU203961, Thailand, Krabi, Talabeng Is., *P. Triboun, s.n. (EDNA09_02286)* (BK); *Paraboea rufescens* (Franch.) B.L.Burtt: JN934772/JN934730

China, Yunnan,19 x 2001, *M. Möller & Y.D. Qi, MMO 01-108/3* (E); *Paraboea siamensis* Triboun: KU203853/KU203948, Thailand, Tak, Umphang, 7 ix 2010, *P. Triboun & al., 4565* (BK, BKF, E); ***Paraboea sinensis* (Oliv.) B.L.Burtt***-*1: **MF358704**/FJ501473, China, Guangxi, Napo, 31 v 2015, *W.B. Xu & J. Guo*, *12352* (IBK)/China, Guangxi Wen He Qun W049 (US 329798) (US); *-*2: **MF358705**/JN934731, China, Guangxi, Fengshan, 2 vi 2015, *W.B. Xu & J. Guo*, *12376* (IBK)/China, Yunnan, Hekou, 20 ix 2006, *M. Möller & L.M. Gao, MMO 06-949b* (E); *-*3: KU203844/KU203939, China, Yunnan, Hekou, 20 ix 2006, *M. Möller & L.M. Gao, MMO 06-949a* (E); ***Paraboea sinovietnamica* W.B.Xu & J.Guo**-1: -/**MF358722**, China, Guangxi, Dahua, 29 iv 2015, *W.B. Xu & al*, *12217* (IBK); *-*2: -/**MF358721**, China, Guangxi, Jingxi, 1 vi 2015, *W.B. Xu & J. Guo*, *12365* (IBK); *-*3: **MF358706/MF358729**, China, Guangxi, Jingxi, 1 vi 2015, *W.B. Xu & J. Guo*, *12366* (IBK); *Paraboea subplana* (B.L.Burtt) C.Puglisi: KU203854/KU203949, Thailand, Krabi, Wat Tham Seua, 11 ix 2008, *D.J. Middleton, 4444* (BK, BKF, E); *Paraboea suffruticosa* (Ridl.) B.L.Burtt: JN934774/JN934732, Thailand, Satun, Mu Ko Phetra National Park, 10 ix 2008, *D.J. Middleton & al., 4432* (BK, BKF, E); *Paraboea swinhoei* (Hance) B.L.Burtt: JN934775/JN934733, China, Guangxi, Xincheng, 23 viii 2006, *M. Möller & Y.G. Wei, MMO 06-783c* (E); *Paraboea tarutaoensis* Z.R.Xu & B.L.Burtt: JN934776/JN934734, Thailand, Satun, ex cult. RBGE20082069, *D.J.Middleton* (E); ***Paraboea tetrabracteata* F.Wen, Xin Hong & Y.G.Wei**: **MF358707/MF358723**, China, Guangdong, Yangchun, 15 iv 2013, *W.B. Xu*, *Pa20130415* (IBK); *Paraboea trachyphylla* Z.R.Xu & B.L.Burtt: JN934777/JN934735, Thailand, Surat Thani, Ban Thakhun, 6 ix 2008, *D.J. Middleton & al., 4310* (E); *Paraboea treubii* (H.O.Forbes) B.L.Burtt: KU203872/KU203967, Indonesia, Sumatra, North Sumatra, 11 vii 2011, *C. Puglisi & al., CP 275* (BO, E); *Paraboea trisepala* W.H.Chen & Y.M.Shui: JN934778/JN934736, China, Guangxi Jingxi, *Y.M. Shui & al., CH153* (KIB); ***Paraboea umbellata* (Drake) B.L.Burtt**: **MF358708/MF358730**, China, Guangxi, Napo, 30 v 2015, *W.B. Xu & J. Guo*, *12340* (IBK); *Paraboea variopila* Z.R.Xu & B.L.Burtt: KU203868/KU203963, Thailand, Nakhon Si Thammarat, Thung Song, 11 ix 2010, *D.J. Middleton & al., 5392* (BK, BKF, E); ***Paraboea velutina* (W.T.Wang et C.Z.Gao) B.L.Burtt-**1: **MF358709/MF358724**, China, Guangxi, Fengshan, 2 vi 2015, *W.B. Xu & J. Guo*, *12375* (IBK); *-*2: **MF358710/ MF358725**, China, Guangxi, Fengshan, 2 vi 2015, *W.B. Xu & J. Guo*, *12375* (IBK); -3: JN934780/JN934738, Guangxi, Fengshan, 4 vi 2007, *M. Möller & Y.G. Wei, MMO 07-1105a* (E); *Paraboea verticillata* (Ridl.) B.L.Burtt: JN934781/JN934739, Malaysia, Selangor, *FRIM, FRI 48225* (KEP); *Paraboea vulpina* Ridl.: JN934782/JN934740, Thailand, Krabi, Muang Krabi, 11 ix 2008, *D.J. Middleton & al., 4442* (E); ***Paraboea xiangguiensis* W.B.Xu & B.Pan**-1: **MF358711/MF358726**, China, Guangxi, Quanzhou, 7 vii 2016, *W.B. Xu & J. Guo*, *13006* (IBK); *-*2: **MF358712/ MF358727**, China, Guangxi, Quanzhou, 7 vii 2016, *W.B. Xu & J. Guo*, *13007* (IBK); *-*3: **MF358713/ MF358728**, China, Guangxi, Quanzhou, 7 vii 2016, *W.B. Xu & J. Guo*, *13008* (IBK).
